# Supplementary material for: Comparative Study of Starch Phosphorylase Genes and Encoded Proteins in Various Monocots and Dicots with Emphasis on Maize
Source: Int J Mol Sci. 2022 Apr 20;23(9):4518. doi: 10.3390/ijms23094518 (PMC9104829; doi:10.3390/ijms23094518)
Supplement: Supplementary file 1 [file ijms-23-04518-s001.zip › ijms-1636137-supplementary.pdf]

Supplementary Material

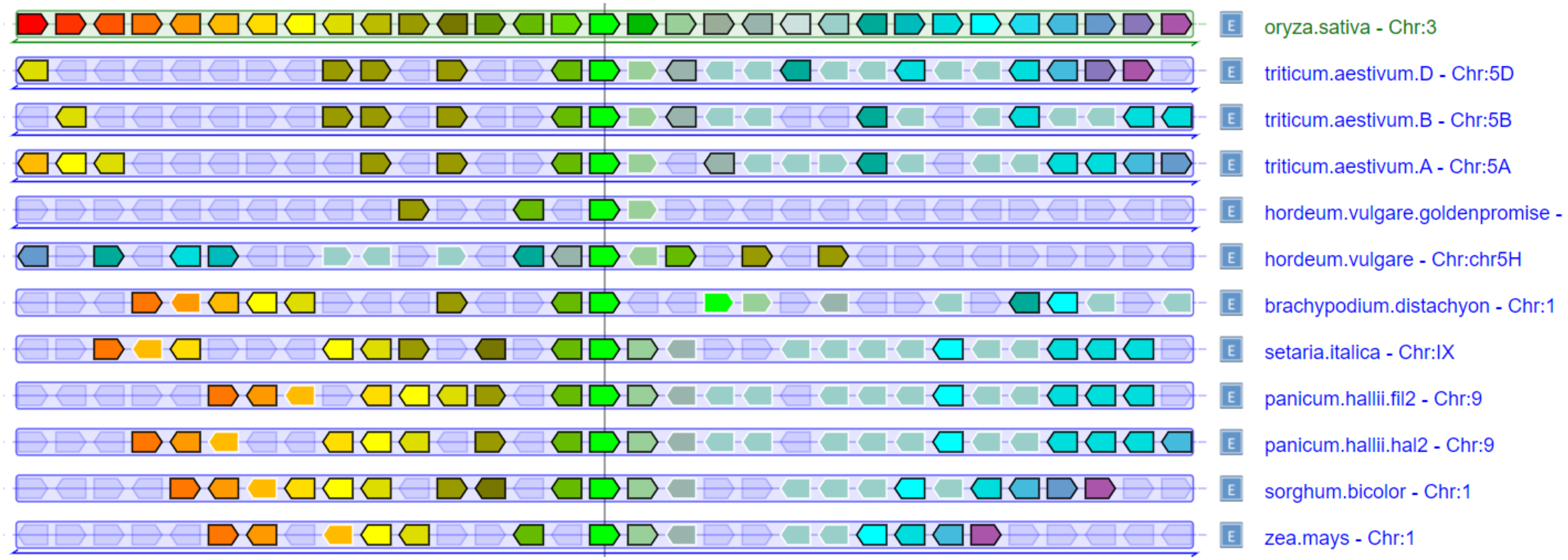

Figure S1. Synteny conservation of *PHO1*.

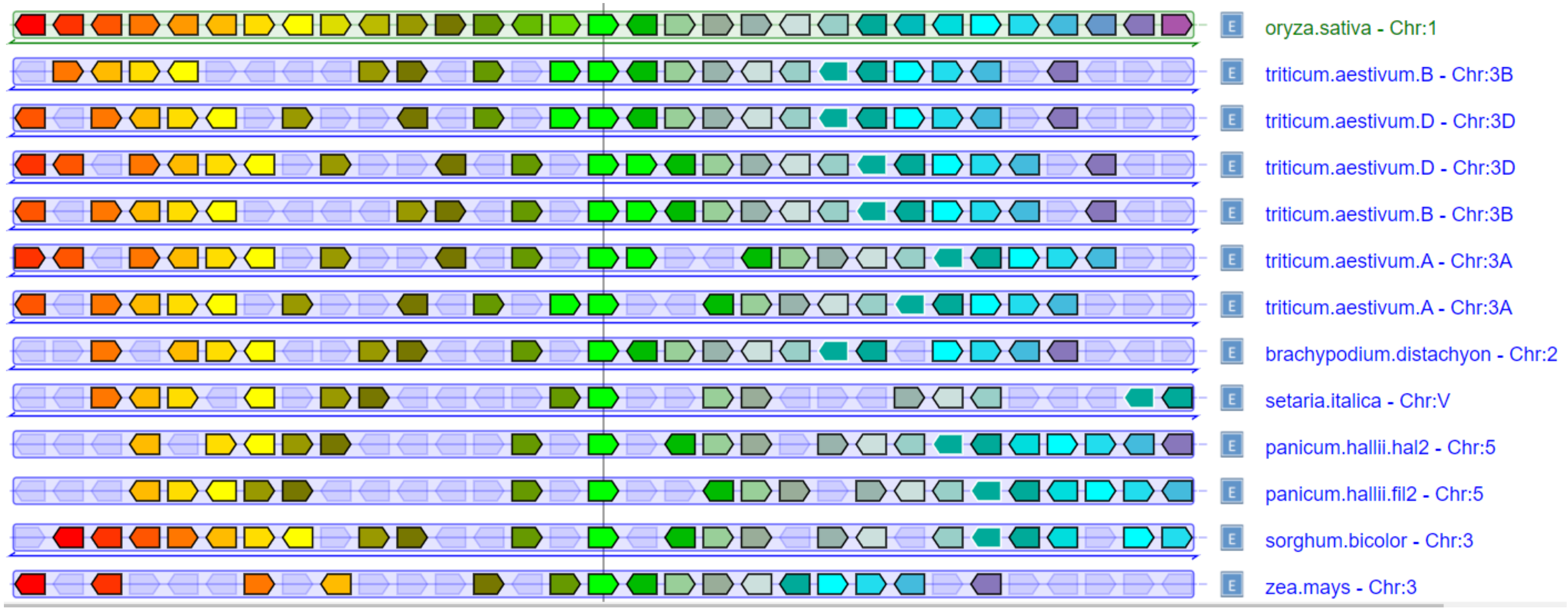

Figure S2. Synteny conservation of PHO2.

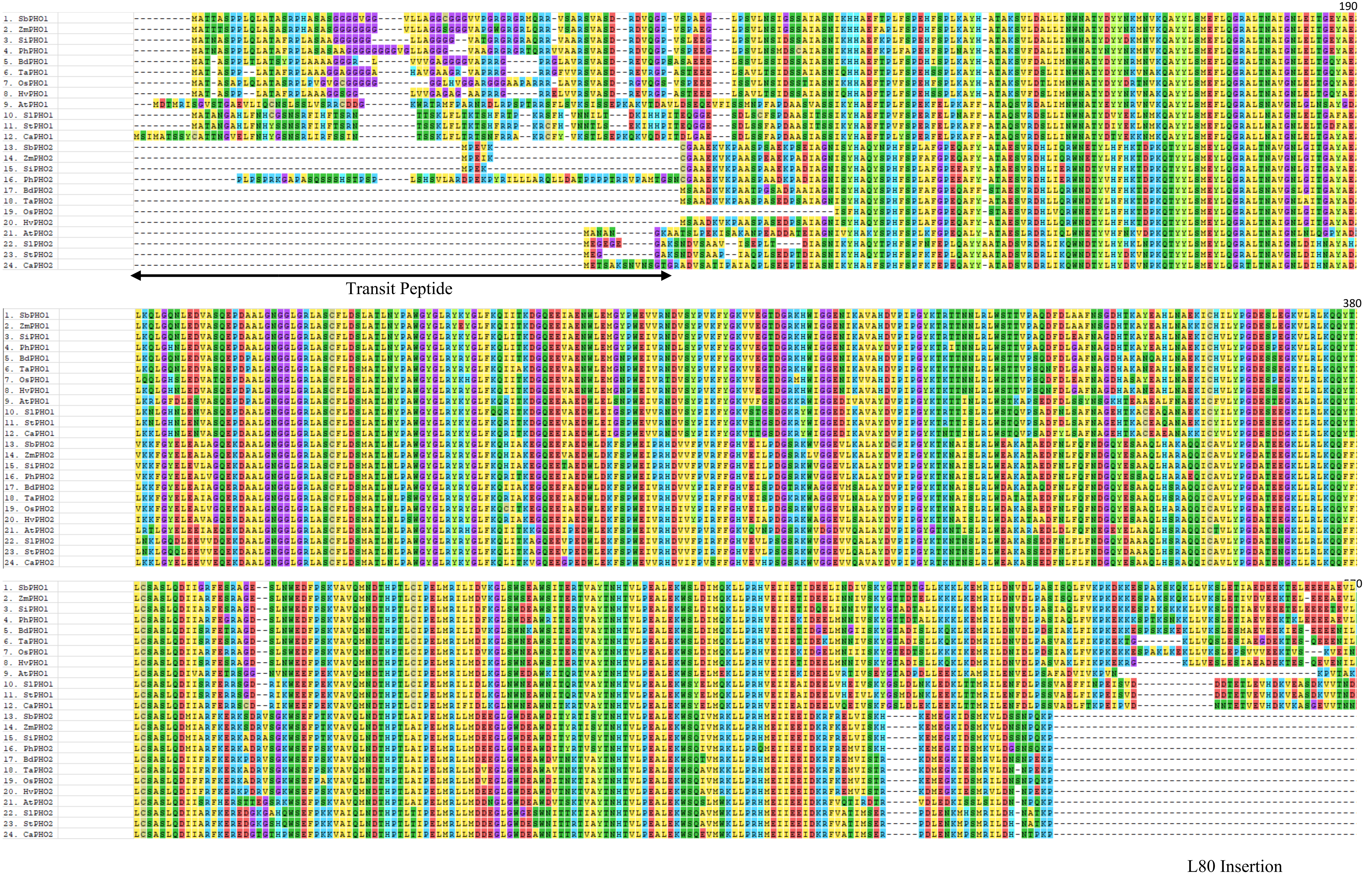
190
1. SbPHO1
2. ZmPHO1
3. SiPHO1
4. PhPHO1
5. BdPHO1
6. TaPHO1
7. OsPHO1
8. HvPHO1
9. AtPHO1
10. SlPHO1
11. StPHO1
12. CaPHO1
13. SbPHO2
14. ZmPHO2
15. SiPHO2
16. PhPHO2
17. BdPHO2
18. TaPHO2
19. OsPHO2
20. HvPHO2
21. AtPHO2
22. SlPHO2
23. StPHO2
24. CaPHO2
Transit Peptide
380
L80 Insertion

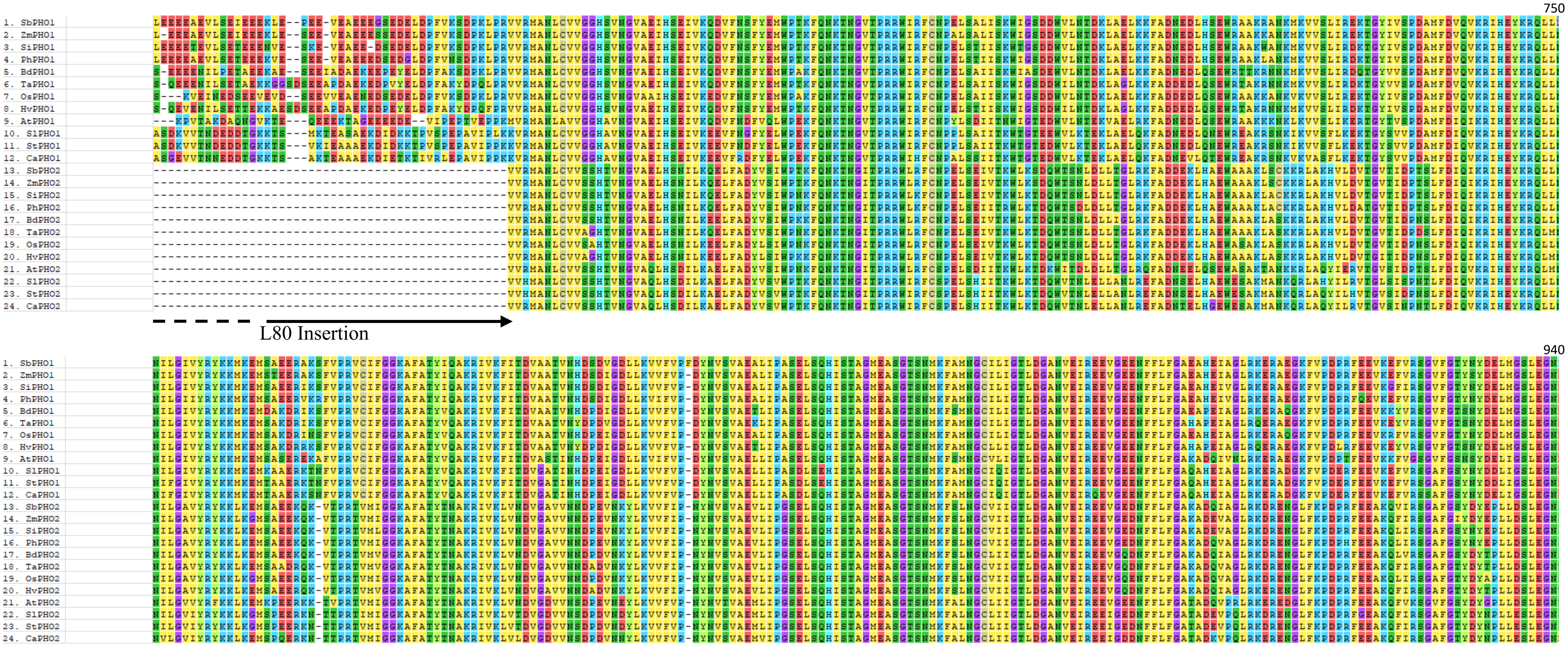
750
1. SbPHO1
2. ZmPHO1
3. SiPHO1
4. PhPHO1
5. BdPHO1
6. TaPHO1
7. OsPHO1
8. HvPHO1
9. AtPHO1
10. SlPHO1
11. StPHO1
12. CaPHO1
13. SbPHO2
14. ZmPHO2
15. SiPHO2
16. PhPHO2
17. BdPHO2
18. TaPHO2
19. OsPHO2
20. HvPHO2
21. AtPHO2
22. SlPHO2
23. StPHO2
24. CaPHO2
L80 Insertion
940

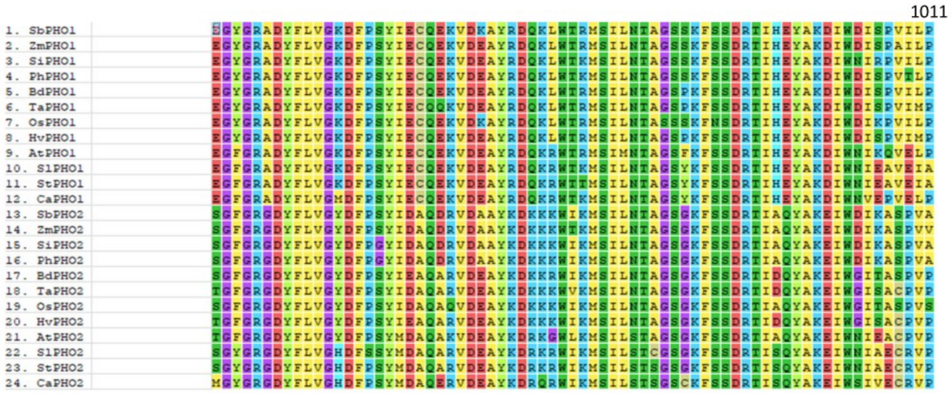

Figure S3. Multiple alignment of PHO sequences in range of species. The sequences of PHO1 include the transit peptide and L80 insertion. Full alignment parts is shown.

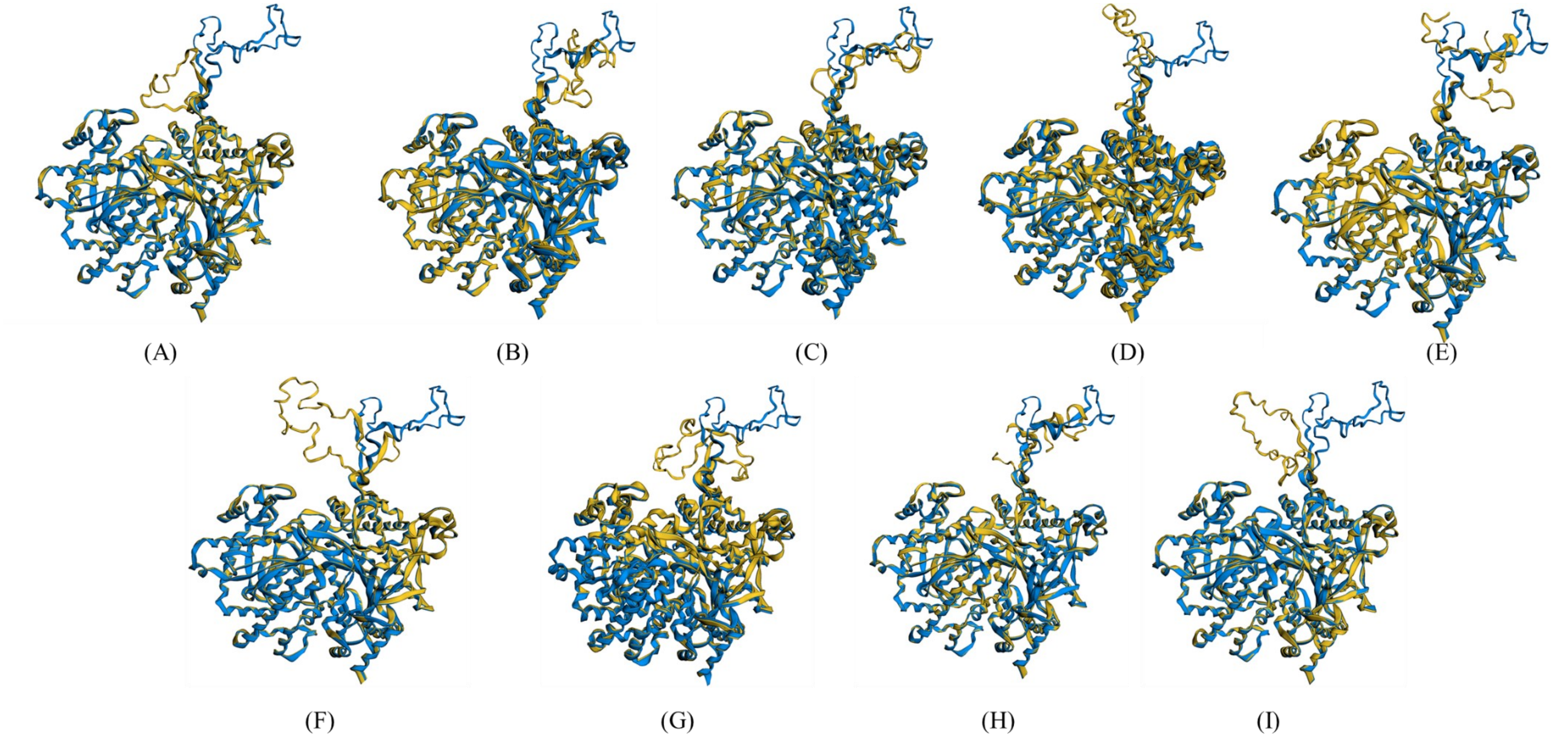

Figure S4. Superimposed structures of the predicted PHO1 (yellow colored) over the *H. vulgare* PHO1 (blue colored) in range of species. (A) AtPHO1. (B) SbPHO1. (C) CaPHO1. (D) OsPHO1. (E) PhPHO1. (F) SiPHO1. (G) SlPHO1. (H) StPHO1. (I) TaPHO1.

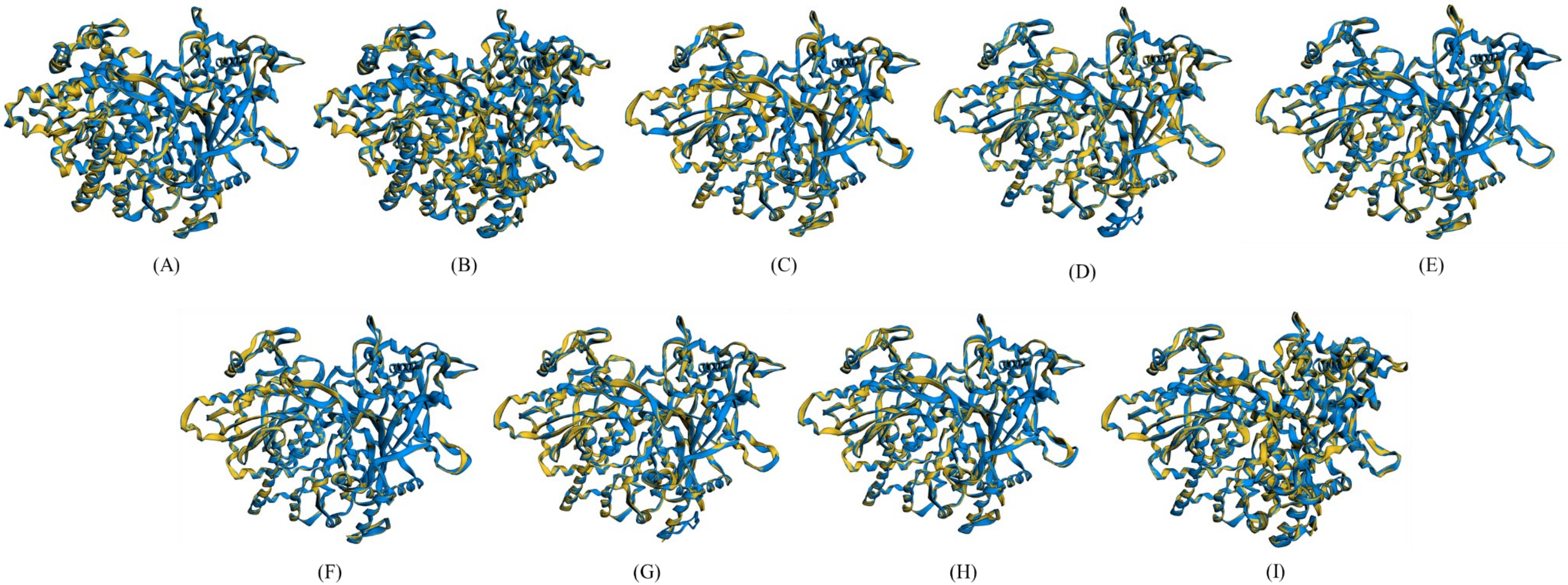

Figure S5. Superimposed structures of the predicted PHO2 (yellow colored) over the *A. thaliana* PHO2 (blue colored) in range of species. (A) AtPHO2. (B) SbPHO2. (C) CaPHO2. (D) OsPHO2. (E) PhPHO2. (F) SiPHO2. (G) SlPHO2. (H) StPHO2. (I) TaPHO2.

Table S1. Percent similarity of exons (upper row) and introns (lower low) of *PHO1* in different species with respect to exons of *PHO1* of *O. sativa*.

**Species**	**Exon/Intron number**														
	**1**	**2**	**3**	**4**	**5**	**6**	**7**	**8**	**9**	**10**	**11**	**12**	**13**	**14**	**15**
Z. mays	52.2	85.0	80.4	86.2	89.1	90.1	89.0	78.3	85.0	89.1	89.0	92.2	93.0	87.1	56.9
	39.6	62.6	47.8	60.0	36.1	56.3	65.2	51.8	52.2	23.8	9.7	36.6	52.5	32.7	-
S. italica	67.5	86.7	86.3	85.4	90.9	88.6	89.0	79.1	85.0	86.8	90.7	90.9	90.9	88.6	59.3
	39.3	41.7	40.3	40.0	44.4	42.8	41.7	38.5	39.7	22.9	39.8	30.0	34.3	44.6	-
P. hallii	67.3	85.4	86.3	86.0	88.8	89.3	90.7	78.3	86.3	83.7	90.3	92.2	91.4	89.1	59.2
	41.1	38.1	40.2	37.2	44.4	44.9	33.7	42.1	40.1	22.8	38.0	37.6	49.6	41.9	-
B. distachyon	65.8	88.4	85.3	88.2	88.4	89.3	90.7	80.3	90.2	86.0	90.3	93.5	92.5	89.6	61.2

	43.0	33.6	43.9	32.9	40.5	34.9	34.3	40.0	37.4	41.8	44.8	44.9	33.6	37.6	-
H. vulgare	64.2	86.4	83.2	86.6	87.4	88.4	90.1	79.2	89.7	85.2	89.1	92.9	91.5	89.2	60.5
	42.3	32.5	41.4	31.6	39.4	33.8	33.2	40.2	38.3	40.5	44.4	43.3	32.5	36.3	-
T. aestivum	63.2	85.4	83.5	85.7	86.3	89.0	90.2	78.4	88.9	85.6	88.2	90.2	89.4	88.6	58.2
	41.3	33.5	40.3	32.3	39.6	34.2	33.5	39.2	39.5	40.1	42.5	42.7	32.8	35.9	-
S. bicolor	60.8	85.8	84.3	86.0	87.7	89.3	90.7	78.4	85.6	87.6	89.9	92.2	92.5	89.1	61.9
	41.0	38.8	41.9	39.3	31.8	43.9	42.0	42.7	38.1	25.0	34.3	35.5	43.5	42.0	-
A. thaliana	45.4	66.7	57.8	76.3	71.4	74.6	80.5	52.4	73.9	77.5	79.8	77.9	77.5	72.6	45.3
	7.3	29.5	28.0	42.7	31.5	42.6	44.0	43.8	31.6	34.8	24.8	28.9	35.3	10.9	-
S. lycopersicum	37.5	65.4	67.6	79.9	71.0	79.0	77.1	56.9	74.5	71.3	78.9	75.3	82.9	79.1	46.2
	41.1	27.7	37.6	32.2	25.1	42.0	43.6	48.2	11.8	35.5	35.4	8.3	37.9	12.5	-
S. tuberosum	34.9	32.1	14.8	44.6	29.9	43.8	32.2	X	X	X	X	X	X	X	X
	16.9	41.2	20.3	9.3	16.1	35.6	-	-	-	-	-	-	-	-	-
C. annuum	33.7	65.7	69.6	79.2	69.6	78.3	77.1	56.1	71.9	71.3	79.7	81.8	82.4	56.8	47.5
	43.0	31.1	38.1	36.0	29.7	30.7	45.3	50.8	13.7	35.8	34.3	20.7	43.4	16.0	-

x indicates absence of exons, - indicates absence of introns

Table S2. Percent similarity of exons (upper row) and introns (lower low) of *PHO2* in different species with respect to exons of *PHO2* in *O. sativa*.

**Species**	**Exon/Intron number**														
	**1**	**2**	**3**	**4**	**5**	**6**	**7**	**8**	**9**	**10**	**11**	**12**	**13**	**14**	**15**
Z. mays	66.8	96.1	88.7	82.6	90.0	92.5	87.0	89.0	82.6	87.2	90.2	85.5	86.7	90.6	52.0
	46.0	13.5	42.0	45.2	27.2	4.9	42.6	36.3	41.1	41.1	35.1	36.7	31.6	44.5	-
S. italica	72.9	92.8	88.7	82.6	90.0	91.4	87.0	89.0	84.8	88.6	91.2	87.4	86.7	90.1	58.1
	62.5	29.4	59.1	50.6	29.5	53.7	59.9	34.9	48.9	70.1	51.0	53.1	32.5	48.8	-
P. hallii	73.1	95.6	91.0	81.4	87.4	90.3	89.7	88.1	82.6	87.7	90.2	87.4	87.9	88.5	55.2
	45.2	29.3	40.6	43.5	21.0	43.4	46.4	35.6	41.5	41.3	39.6	43.6	34.2	19.5	-
B. distachyon	66.9	92.2	86.4	87.2	88.4	91.4	88.6	89.0	89.4	90.4	92.6	87.4	90.5	89.5	62.7
	58.6	32.9	59.1	53.6	57.9	54.1	48.0	39.9	23.5	70.9	55.9	55.6	60.3	45.9	-
H. vulgare	65.4	91.4	86.3	88.2	87.6	90.7	87.2	88.6	88.1	89.7	91.2	86.3	89.7	89.2	61.6
	46.2	33.2	58.2	50.2	56.2	53.8	47.2	38.8	24.2	70.4	53.2	53.7	58.9	52.0	-
T. aestivum	62.8	90.5	87.5	81.2	89.1	89.4	85.2	82.1	80.6	86.7	90.1	84.2	86.2	89.0	52.3
	43.0	14.2	45.6	42.3	26.7	52.4	46.3	37.2	40.5	67.5	49.1	45.6	34.9	41.6	-
S. bicolor	73.8	96.1	88.1	80.2	87.9	92.5	88.1	91.5	84.1	87.2	89.7	86.8	85.6	91.1	53.9

	53.5	29.3	56.7	47.5	29.2	52.0	56.1	34.9	47.9	67.8	48.9	49.0	34.0	42.6	-
A. thaliana	38.0	42.3	42.7	29.3	28.4	34.4	30.6	37.6	42.7	35.3	44.7	39.9	35.3	40.4	23.7
	35.4	32.3	44.8	10.8	23.3	22.5	39.0	24.4	19.7	38.1	43.2	44.1	40.7	26.2	=
S. lycopersicum	26.8	43.2	43.8	29.0	27.3	31.6	30.6	34.0	44.1	35.1	41.9	40.8	32.5	39.2	23.7
	33.5	17.7	20.4	11.3	23.3	27.7	16.8	47.8	24.0	11.7	46.8	45.4	1.8	26.4	=
S. tuberosum	16.9	40.3	46.2	29.2	27.3	32.3	33.4	36.0	43.4	34.5	42.3	40.8	34.0	39.6	23.3
	38.1	31.2	5.2	11.4	24.6	28.7	6.5	43.8	23.6	10.0	42.4	45.1	3.3	25.4	=
C. annuum	31.1	35.4	46.1	28.8	29.7	34.6	29.6	33.5	42.7	37.9	43.9	42.7	32.2	39.3	23.8
	28.8	16.8	1.0	10.8	17.3	27.9	9.6	45.2	23.4	9.7	5.8	45.8	1.0	25.9	=

x indicates absence of exons, - indicates absence of introns, = have intron but did not analyze

Table S3. Simple sequence repeats (SSRs) and retro-elements identified in *PHO1*.

Species	Position in bp	Type	Repeat	Size(bp)
S. bicolor	147-199	SSR	(CGC)n	50
	1665-1739	SSR	(TTCTATT)n	45
Z. mays	183-239	SSR	(CGC)n	52
	4442-4480	SSR	(AAGAGCA)n	41
	6512-6558	SSR	(ATCTT)n	48
S. italica	8-49	SSR	(CATCG)n	46
	90-138	SSR	(CGC)n	45
P.hallii	77-136	SSR	(CGCCTC)n	60
	138-162	SSR	(GGGGGC)n	25
	2633-2719	SSR	(ATGTTT)n	89
B. distachyon	104-163	SSR	(CCG)n	60
O. sativa	26-63	SSR	(CT)n	38
	150-171	SSR	(CGGCGG)n	122
	407-436	SSR	(CG)n	30
	5138-5171	SSR	(TCTTCA)n	33
A. thaliana	2621-2667	Low-complexity	GA-rich	47
C. annuum	3128-3197	SSR	(TTCTT)n	64
	7350-7407	SSR	(TTTTCT)n	57

Table S4. Simple sequence repeats (SSRs) and retro-elements identified in *PHO2*.

Species	Position in bp	Type	Repeat	Size(bp)
S. bicolor	5917-5940	SSR	(T)n	24
S. italica	1-33	SSR	(TCC)	30
P.hallii	45-77	SSR	(CTCCCA)	33
B. distachyon	3812-3836	SSR	(TTTA)n	26
O. sativa	31-62	SSR	(CCGTT)n	32
A. thaliana	225-256	Low-complexity	A-rich	31
	849-887	SSR	(TG)n	
S. lycopersicum	5122-5168	SSR	(AATTTAC)n	45
	8035-8068	SSR	(TATTCT)n	34
	11669-11703	SSR	(CTATTC)n	35
S. tuberosum	141-169	SSR	(TCTTT)n	28
	2048-2082	SSR	(TTATTT)n	33
	5826-5846	SSR	(TG)n	21
	6987-7033	SSR	(AATTTAC)n	45
C. annuum	3543-3597	SSR	(CAA)n	54
	1849418541	Low-complexity	G-rich	47
	18620-18670	SSR	(ATTCA)n	49
	31248-31296	SSR	(CAA)n	45
	31355-31402	Low-complexity	A-rich	50
	32006-32087	Low-complexity	GA-rich	77

Table S5. Details of 3D structure and quality assessment parameters of PHO1 (oligostate-homodimer) (upper row) and PHO2 (oligostate-homodimer; lower row) in all of the monocots and dicots obtained using Swiss-Model.

Species	Template used	% Identity	GMQE score	Quality assessment parameters	
				QMEAN 6 score	Dfire energy
S. bicolor	5lr8.1.A	86.98	0.78	0.88	-687.17
	4bqe.1.A	79.44	0.91	0.89	-2712.1
Z. mays	5lr8.1.A	87.51	0.79	0.89	-578.17
	4bqe.1.A	79.30	0.91	0.89	-2617.12
S. italica	5lr8.1.A	87.25	0.79	0.89	-581.25
	4bqe.1.A	79.18	0.91	0.89	-2812.12

P. hallii	5lr8.1.A	87.00	0.78	0.88	-612.21
	4bqe.1.A	79.20	0.83	0.90	-2632.12
B. distachyon	5lr8.1.A	91.96	0.80	0.90	-674.23
	4bqe.1.A	79.42	0.92	0.89	-2576.12
T. aestivum	5lr8.1.A	97.62	0.82	0.91	-643.12
	4bqe.1.A	80.24	0.92	0.90	-2721.15
O. sativa	5lr8.1.A	86.10	0.79	0.89	-674.12
	4bqe.1.A	80.92	0.93	0.90	-2671.32
A. thaliana	5lr8.1.A	76.43	0.77	0.88	-587.12
	4bqe.1.A	100	0.95	0.93	-2442.31
S. lycopersicum	5lr8.1.A	74.34	0.78	0.88	-567.43
	4bqe.1.A	81.33	0.90	0.90	-2521.45
S. tuberosum	5lr8.1.A	74.70	0.78	0.87	-589.32
	4bqe.1.A	81.43	0.91	0.90	-3489.52
C. annuum	5lr8.1.A	74.69	0.76	0.87	-598.23
	4bqe.1.A	80.36	0.91	0.90	-2563.27

Table S6. Model quality assessment scores of 3D structures of PHO1 (upper row) and PHO2 (lower row) in 11 species.

**Species**	**PROCHEK**					**ERRAT**	**VERIFY3D**	**Status of 3D structure from SAVES**
	**Favoured* region (%)**	**Allowed region (%)**	**Generously allowed region (%)**	**Disallowed region (%)**	**G-factor**	**Quality factor (%)**	**(3D-1D Profile) (%)**	
S. bicolor	91.1	7.7	0.7	0.5	-0.11	95.19	0.2	PASS
	91.5	8.2	0.3	0.0	-0.04	95.88	0.2	PASS
Z. mays	90.4	8.3	0.8	0.6	-0.12	96.34	0.2	PASS
	91.5	8.2	0.3	0.0	0.00	96.62	0.2	PASS
S. italica	90.1	8.4	1.0	0.6	-0.11	97.70	0.2	PASS
	91.4	8.2	0.3	0.1	-0.03	96.19	0.2	PASS
P. hallii	90.4	7.7	1.1	0.7	-0.11	96.76	0.2	PASS
	91.5	8.2	0.3	0.0	0.00	96.62	0.2	PASS
B. distachyon	89.5	9.0	0.7	0.7	-0.07	96.20	0.2	PASS
	91.3	8.4	0.3	0.0	0.01s	95.34	0.2	PASS
T. aestivum	91.0	7.2	1.1	0.6	-0.12	96.82	0.2	PASS
	91.3	84	0.3	0.0	0.00	96.80	0.2	PASS

O. sativa	90.8	7.8	0.9	0.5	-0.10	95.87	0.2	PASS
	91.8	7.9	0.3	0.0	-0.02	96.63	0.2	PASS
A. thaliana	91.5	7.4	0.6	0.4	-0.09	97.06	0.2	PASS
	91.3	8.4	0.3	0.0	0.01	97.61	0.2	PASS
S. lycopersicum	91.4	7.6	0.7	0.3	-0.08	94.71	0.2	PASS
	91.8	7.9	0.2	0.0	-0.04	96.63	0.2	PASS
S. tuberosum	91.2	7.5	0.9	0.4	-0.10	92.87	0.2	PASS
	91.8	7.9	0.3	0.0	0.00	95.77	0.2	PASS
C. annuum	90.9	7.4	1.1	0.6	-0.09	96.45	0.2	PASS
	92.2	7.6	0.2	0.0	-0.04	96.26	0.2	PASS

* Based on an analysis of 118 structures of resolution of at least 2.0 A° and R-factor no greater than 20%, a good quality model would be expected to have maximum number of amino acids in the most favoured regions.

Table S7. Predicted values of different parameters of superimposition of 3D protein structures of PHO1 and PHO2 of different plant species over 3D protein structure of *H. vulgare* PHO1 and *A. thaliana* PHO2 respectively.

**Species**	**RMSD***		**%Identity**		**%Similarity**	
	**PHO1**	**PHO2**	**PHO1**	**PHO2**	**PHO1**	**PHO2**
S. bicolor	5.68	0.04	92.82	97.94	94.34	98.91
Z. mays	2.74	0.21	89.10	96.98	91.59	99.27
S. italica	2.04	0.06	86.29	95.53	88.95	98.55
P. hallii	2.42	1.63	86.65	56.56	91.68	70.45
B. distachyon	3.12	0.05	84.46	92.82	89.89	97.15
T. aestivum	3.62	0.07	83.14	90.55	88.83	95.88
O. sativa	2.20	0.25	73.73	79.32	85.54	90.33
A. thaliana	3.87	0.34	74.83	78.40	85.87	89.20
S. lycopersicum	2.10	0.26	74.70	78.67	86.41	89.21
S. tuberosum	2.41	0.37	74.09	77.27	85.23	89.12
C. annuum	5.68	0.04	92.82	97.94	94.34	98.91

*Root Mean Square Deviation.

Table S8. Ligand Binding amino acid residues and their positions in PHO1 and PHO2.

Species	Amino acids and their position															
	PHO1															
S. bicolor	L	G	G	R	W	V	K	Y	N	V	A	G	T	S	N	K
	149	193	194	197	637	714	715	799	800	801	804	826	827	828	829	831
Z. mays	L	G	G	R	W	V	K	Y	N	V	A	G	T	S	N	K
	149	193	194	197	636	713	714	798	799	800	803	825	826	827	828	830
S. italica	L	G	G	R	W	V	K	Y	N	V	A	G	T	S	N	K
	145	189	190	193	632	709	710	794	795	796	799	821	822	823	824	826
P. hallii	L	G	G	R	W	V	K	Y	N	V	A	G	T	S	N	K
	151	195	196	199	639	716	717	801	802	803	806	828	829	830	831	833
T. aestivum	L	G	G	R	W	V	K	Y	N	V	A	G	T	S	N	K
	140	184	185	188	623	700	701	785	786	787	790	812	813	814	815	817
O. sativa	L	G	G	R	W	V	K	Y	N	V	A	G	T	S	N	K
	144	188	189	192	630	707	708	792	793	794	797	819	820	821	822	824
A. thaliana	L	G	G	R	W	K	Y	N	V	A	G	T	S	N	K	
	159	203	204	207	614	692	776	777	778	781	803	804	805	806	808	
H. vulgare	L	G	G	R	W	V	K	Y	N	V	A	G	T	S	N	K
	137	181	182	185	620	697	698	782	783	784	787	809	810	811	812	814
	PHO2															
C. annuum	L	G	G	R	W	K	Y	N	V	A	G	T	S	N	K	L
	99	143	144	147	500	578	661	662	663	666	688	689	690	691	693	99

Note: Missing species are not identified to posses the ligand binding sites
